# Is Unfavourable Cervix prior to Labor Induction Risk for Adverse Obstetrical Outcome in Time of Universal Ripening Agents Usage? Single Center Retrospective Observational Study

**DOI:** 10.1155/2020/4985693

**Published:** 2020-09-01

**Authors:** Mlodawski Jakub, Mlodawska Marta, Galuszewska Jagoda, Glijer Kamila, Gluszek Stanislaw

**Affiliations:** ^1^Collegium Medicum, Jan Kochanowski University in Kielce, Poland; ^2^Eskulap, Student Scientific Organization–Collegium Medicum, Jan Kochanowski University in Kielce, Poland

## Abstract

Cervical assessment on the Bishop scale prior to induction of labor (IOL) is one of the strongest prognostic criteria in relation to the success of the procedure. The commonly used preinduction methods are mainly aimed at reducing the percentage of cesarean sections. Our study has analyzed obstetric results of patients who had unripe cervix (Bishop score <7) before IOL and used preinduction (Foley catheter or misoprostol vaginal insert releasing 7 mcg of misoprostol per hour for 24 hours) with obstetric results of patients in whom, due to favourable cervix, only a low-dose infusion of oxytocin was used. We reviewed the medical records of 1010 single pregnancies in whom IOL was performed. We divided the patients into two groups: group A (where preinduction was used) and group B (Bishop score ≥7 points) where preinduction was not used. Patients in group A were more likely to complete the delivery by caesarean section (OR = 4.58, 95% CI 3.22-6.51), and more likely to have events that were indications for operative delivery: unreassuring fetal heart rate trace (OR = 3.29, 95% CI 2.07-5.23) and arrested labor or failed induction (OR = 3.4, 95% CI 2.06-5.62). The groups did not differ in the percentage of vacuum extraction, postpartum haemorrhage, and meconium stained amniotic fluid. In group B, more infants were born with umbilical cord blood pH <7.1 (1.38% vs. 0%), both groups included no deliveries of newborns with Apgar score ≤3 points, the groups did not differ in terms of the percentage of newborns with Apgar score between 4 and 7 at birth (OR = 0.66, 95% CI 0.29-1.49). The immature cervix and the need to use labor preinduction is a risk factor for caesarean section. The necessity of preinduction does not impair neonatological results.

## 1. Introduction

Induction of labor (IOL) is one of the most common procedures performed in modern obstetrics. In countries with a high level of economic development, approximately 20% of pregnant women and 30-40% of patients who give birth vaginally are affected by IOL [1, 2]. Part of the patients who, on medical grounds, qualify for delivery induction has unripe cervix (usually defined as a Bishop score ≤6). Prior to induction with an oxytocin infusion, the total Bishop score and its individual elements (such as fetal station, cervical length/effacement, cervical position or consistency, and dilatation) are a key element of most predictive models that estimate the risk of ineffective induction [3]. Therefore, patients with low scores have an indication for the use of mechanical or biochemical preinduction. Currently, many methods of delivery preinduction exist, and the prevalence of their usage varies considerably between countries. The use of preinduction reduces the risk of cervical dystocia and ineffective IOL using only oxytocin [2]. In our study, we decided to analyze the obstetric results of patients who were subjected to a preinduction procedure prior to delivery compared to patients with ripe cervix (Bishop score ≥7) who did not require preinduction.

## 2. Objectives

The aim of the study is to assess whether the use of preinduction and a low Bishop scale score (≤7) is a risk factor for adverse obstetric outcomes.

## 3. Material and Methods

It was a retrospective observational study. We reviewed medical records of 1010 patients who delivered in the Department of Gynecology and Obstetrics of the Provincial Hospital Complex in Kielce (tertiary referral hospital) in the period from 01.02.2018 to 1.07.2019. All included patients were in a singleton pregnancy, the fetus was in the cephalic presentation, and the gestational age was greater than 37 weeks. All deliveries were induced. The deliveries were induced from medical indications according to the recommendations of the Polish Society of Gynecology and Obstetrics [1]. The amount of patients included in the study was calculated based on prevalence of newborns born with Apgar score ≤7 in our center in the year preceding the study and the estimated prevalence difference between the groups of 1% (based on unpublished preliminary study) at the preset parameters (power = 80%, alpha significance level = 0.05). We divided the patients into two groups—patients who had preinduction of labor (cervical maturity on the Bishop scale <7) (group A) (*n* = 503), and patients in whom the cervix was rated >6 points on the Bishop scale (group B) (*n* = 507), and only oxytocin was used for labor induction. Patients in group A were preinduced with a 20F size Foley catheter with balloon-filled up to 60 ml of saline (368 patients) or with misoprostol vaginal insert [MVI] 200 *μ*g released 7 *μ*g/h for 24 hours (Misodel ® Ferring Pharmaceuticals Poland) (135 patients). Preinduction was removed after a maximum of 24 hours or in case of spontaneous delivery onset defined as cervical dilation ≥4 cm with regular uterine contractions. Patients in whom the preinduction was removed for other reasons were excluded. Oxytocin in the induction of labor was used in the low dose protocol [4]. We compared the groups in terms of obstetric results—the percentage of operative deliveries (cesarean sections (CS) and vacuum extraction (VE)) and the most common indications for these procedures, the occurrence of postpartum haemorrhage (PPH), and meconium-stained amniotic fluid (MSAF). We also compared the clinical condition of newborn based on Apgar score and pH from venous cord blood. We performed statistical analysis using Statistica 13.1 (StatSoft Poland). In case of continuous variables, we presented the arithmetic mean when the distribution was close to normal and the median in case of skewed distribution as a measure of central tendency. Standard deviation and interquartile range were used as measures of scatter, respectively. We compared the groups in the case of near-normal distribution and equal variance assumptions with the Student's *t*-test, and in the case of failure to meet the abovementioned criteria, we compared the groups with the Kruskal-Wallis *U* test. In the case of qualitative variables, we presented the data as a percentage of events in a given group, we compared the groups using the Pearson's *χ*^2^ test, and in the case of small expected numbers, we applied the Yates correction.

## 4. Results

The demographic characteristics of the groups are presented in Table 1. The percentage of pluriparous women and gestational age at delivery did not differ significantly between the groups. In the group B, epidural analgesia was used statistically more often. The comparison of the studied groups is presented in Table 2. The percentage of CS was significantly different between the groups. The chance of CS delivery was 4.58 times higher (95% CI 3.22-6.51) in A group. The groups did not differ in the percentage of births completed by VE. The most common indication for operative delivery was unreassuring fetal heart rate tracing, which was significantly more frequent in group A patients (OR = 3.29, 95% CI 2.07-5.23). In group A, arrested labor or failure IOL was significantly more frequent as an indication for CS. The groups did not differ significantly in the occurrence of PPH and MSAF. The median pH of umbilical cord blood in group B was significantly lower; although, both values were within normal range. The percentage of newborns born with pH of venous cord blood <7.2 did not differ between groups. Significantly more often children with pH <7.1 were born in group B. The group of preinduced patients included no children in whom the pH value would be lower than 7.1. The pH cut-off values used in the comparison were selected on the basis of literature data indicating an increasing risk of neonatal morbidity and mortality at the specified values [5, 6].

In none of the groups, a newborn with Apgar score 3 or less was born. Clinical status of newborns expressed as percentage of general medium condition (Apgar 3-7 points) did not differ between groups. Both groups did not differ in terms of the time patients spent in the delivery room.

## 5. Discussion

Preinduction of delivery before the start of IOL with oxytocin is performed for several reasons. Favourable cervix before oxytocin infusion reduces the number of ineffective IOL and the need for requalification for IOL [1, 2]. Higher Bishop score and shorter cervix in vaginal ultrasound also reduces the percentage of cesarean sections and is associated with lower maternal and neonatal morbidity and shorter hospitalization time [2, 7, 8]. Such procedures also have a strong physiological basis. The cervix plays an extremely important role in generating intrauterine pressure. The shape of the uterus is a sphere with a fixed radius, and the pressure within the uterine cavity can be described by Laplace's law, according to which the pressure generated inside is proportional to twice the tension of the walls and inversely proportional to the radius of the sphere (*P* = 2*T*/*R* where *T*is the tension and*R*is the radius of sphere) [9]. Due to the asymmetric distribution of the uterine muscles, pressure is generated mainly in the area of the fundus, and the possibility of “pulling” the cervical edges makes it possible to limit the value of maximum intrauterine pressure. The latter is closely related to the favourability of the cervix. Low cervical susceptibility (lower Bishop score) results in the generation of higher intrauterine pressures. Pressure during contraction also increases more rapidly. In the model developed by Gee et al. [9], the intrauterine pressure is approx. 1.5 times higher for the same wall tension at a 3 cm long cervix compared to a 0.5 cm long cervix. This phenomenon has extremely important clinical implications. The administration of oxytocin inducing systolic activity at the immature cervix increases muscle tension and generates higher intrauterine pressures, which may translate into impaired umbilical cord blood flow, cause fetal distress, and increase the risk of uterine muscle rupture. For this reason, induction of delivery after cesarean section in the case of unripe cervix is associated with a four-fold higher risk of uterine rupture in the case of uterine contraction after oxytocin infusion (hazard ratio [HR] = 4.09, 95% CI 1.82-9.17) [10]. These elements imply a modern approach for IOL based on the infusion of oxytocin only after cervical ripening. The data collected in our study show that despite the use of preinduction in patients, spontaneous cervical maturation and a high Bishop score at admission is still a positive prognostic factor for the vaginal delivery. Literature data show that a spontaneously started delivery is the most likely to be completed vaginally [11]. In the cited study, the multivariable logistic regression model calculated by the authors indicates that in addition to obesity of the patient, fetal macrosomy, and the age of over 35 years, the assessment of the cervix on the Bishop scale <6 is the greatest risk factor for CS in both spontaneous and induced deliveries. Despite differences in the percentage of CS rates in three groups distinguished in the study: elective, medical-induced, and spontaneously started deliveries (percentage of CS 23.4%, 23.8%, 12%, respectively) after adjusting for the Bishop score at admission, no significant differences in CS rates were found among these 3 groups [11]. In our study, patients who have a cervical score of 6 or less on the Bishop scale at the time of the IOL decision are at four times the risk of CS (OR = 4.58, CI 95% 3.22-6.51), and the main indication is an abnormal cardiotocographic fetal trace.

For many years, the obstetricians have expressed the concern that one of the risk factors of cesarean section in the group of patients undergoing IOL is gestational age less than 41 weeks. After the publication of the ARRIVE study [12] (whose authors proved that induction of birth after the 39th week of pregnancy does not increase the percentage of CS compared to the expectant management), the assessment of the cervix at admission becomes an even more important predictive element in relation to vaginal delivery (due to marginalization of gestational age). Earlier induction of labor in patients with clinical data (such as low Bishop score or long cervix in ultrasonographic cervical measurement) [7] arguing for a low chance of spontaneous birth may reduce the number of maternal complications [11].

In our study, we did not consider the differences between different methods of delivery preinduction, but we only focused on the population effect of their use. Group A was heterogeneous in terms of the methods used. The type of preinduction may also affect perinatal results. In this study, PGE1 prostaglandin (misoprostol) was used as a vaginal insert with constant release for 24 hours. PGE1 used vaginally is associated with higher risk of uterine hyperstimulation with fetal FHR changes compared to prostaglandin PGE2 (dinoprostone) (RR = 1.75, 95% CI 1.14-2.78) [13], which may increase the percentage of CS when using the former. The Foley catheter used in our study, whose history dates back to the 19th century, is also not the only mechanical method used in cervical ripening. It seems that Cook's balloon catheter, invented in the early 90's, may be more effective in preinduction cervical ripening compared to Foley's catheter and PGE2 prostaglandin [14, 15]. Studies indicate that the combination of mechanical method with vaginal prostaglandins does not bring additional benefits such as reduction time to vaginal delivery or reduction in cesarean section rate [16].

Minimising the risk of hyperstimulation is a basic advantage of mechanical methods in delivery preinduction, it enables their use also in outpatient settings, and current evidence available in the literature indicates the safety of such a procedure [17]. This is important at a time when an increasing number of patients in developed countries are interested in reducing the medicalisation of delivery and renouncing deliveries in hospital wards in favour of birth houses or own home's birth [18].

In our cohort, the most common reason for CS was an abnormal CTG trace. The probability of this complication was 3.29 times higher in the group of preinduced patients but did not translate into worse neonatal results in the group of preinduced patients; on the contrary, the percentage of newborns born with pH <7.1 is significantly higher in the group of patients induced only with oxytocin. The question that remains unresolved is the following: “did the better outcomes of cord blood acid-base balance result from a higher percentage of surgical deliveries?” The nature of this work does not allow to answer such a question. At the same time, one should remember that the cardiotocographic record has false positive (false pathological) changes in up to 50% [19], and seemingly, pathological changes for the person supervising the delivery may in fact be physiological changes in the fetus' heart rate. All deliveries in the study were subject to continuous CTG monitoring, because induced deliveries are not physiological in the meaning of Polish law [20]. The confounding factor in this study may be related to the difference in the percentage of epidural analgesia (EA) between groups. Some literature data indicate that EA correlates with increases neonatal care unit admission of newborns (OR 1.89, 95% CI 1.45 to 2.46), respiratory distress (OR 1.49, 95% CI 1.07 to 2.07) and need for oxygen in first day of life (OR 1.44, 95% CI 1.01 to 2.07) [21]. However, literature data do not indicate an increased risk of acidosis in children born during deliveries with EA [22].

## 6. Conclusion

The immature cervix and the need for delivery pre-induction is a risk factor for cesarean sections. The need for preinduction does not impair neonatological results.

## Figures and Tables

**Table 1:** Demographic characteristics of groups.

	Group A (*n* = 503)	Group B (*n* = 507)	*p* value
Age (years)	28.14 (SD = 4.59)	28.32 (SD = 5.0)	*p* = 0.07
Gestational age at delivery (weeks)(median, IQR)	40 (0.8)	40 (0.9)	*p* = 0.92
Pluripara	20.08%	24.26%	*p* = 0.1
Epidural analgesia	19.28%	27.42%	*p* = 0.002

**Table 2:** Comparison of groups.

	Group A (*n* = 503)	Group B (*n* = 507)	*p* value	OR (95% CI)
Time at delivery room (h)(SD)	8.31 (6)	8.66 (4.8)	0.06	N/A
Cesarean section	32.41%	9.47%	*p* ≤ 0.001	4.58 (3.22-6.51)
Unreassuring fetal CTG	14.60%	4.78%	*p* ≤ 0.001	3.29 (2.07-5.23)
Arrested labor and induction failure	15.11%	5.13%	*p* ≤ 0.001	3.4 (2.06-5.62)
Vacuum extraction	2.58%	3.35%	*p* = 0.47	0.63 (0.47-1.84)
Postpartum haemorrhage	2.19%	0.99%	*p* = 0.12	0.76 (0.36-1.59)
Meconium stained amniotic fluid	12.52%	8.09%	*p* = 0.02	1.62 (1.07-2.46)
pH (median, IQR)	7.36 (0.07)	7.35 (0.09)	*p* = 0.001	N/A
pH <7.2	2.58%	4.14%	*p* = 0.17	0.61 (0.3-1.24)
pH <7.1	0%	1.38%	*p* = 0.008	N/A
Apgar 4-7 points	1.99%	2.96%	*p* = 0.32	0.66 (0.29-1.49)

## Data Availability

The data that support the findings of this study are openly available in OSF Storage at doi:10.17605/OSF.IO/N6W4G.
